# Intraoperative diagnosis of an unsuspected ruptured left ventricular aneurysm using transesophageal echocardiography: A case report

**DOI:** 10.1002/ccr3.1902

**Published:** 2018-11-11

**Authors:** Tsuyoshi Tagawa, Shigeki Sakuraba

**Affiliations:** ^1^ Division of Clinical Anesthesia Mie University Hospital Tsu Japan; ^2^ Department of Anesthesiology Clinical Care Medicine Kanagawa Dental College Kanagawa Japan

**Keywords:** left ventricular aneurysm, myocardial infarction, transesophageal echocardiography

## Abstract

Transesophageal echocardiography (TEE) enables a more accurate visualization of left ventricular posterior aneurysms than transthoracic echocardiography due to the close proximity of the esophagus to the posterior ventricular wall. Therefore, TEE is essential for the accurate diagnosis of posterior aneurysm, particularly in urgent settings where preoperative assessments may be insufficient.

## 1 INTRODUCTION

Left ventricular (LV) aneurysm in posterior locations is rare and relatively benign, being associated with only a small risk of rupture. However, once cardiac rupture occurs, it can cause catastrophic hemodynamic compromise. Transthoracic echocardiography (TTE) has a key role in the early diagnosis and prompt management of patients with cardiac rupture. Here, we describe the utility of intraoperative transesophageal echocardiography (TEE) in diagnosing a ruptured LV aneurysm that was missed on preoperative examination with TTE.

## 2 CASE REPORT

**Figure 1:**
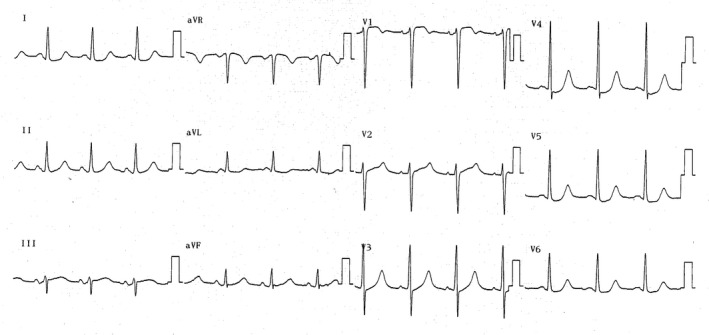
Electrocardiography revealed a normal sinus rhythm with mild ST‐segment elevation in V1 and depression in V3‐V6

A 55‐year‐old patient with a past medical history of hypertension and type 2 diabetes mellitus was transferred to our institution with a sudden onset of chest pain. He had a medication history of metoprolol for hypertension. In the emergency room, he was pale and diaphoretic with a heart rate of 62 beats/min and a blood pressure of 76/48 mm Hg. He showed no difference in blood pressure among the limbs. Electrocardiography revealed a normal sinus rhythm with mild ST‐segment elevation in V1 and depression in V3‐V6 (Figure 1), and chest X‐ray demonstrated an increased cardiac silhouette. TTE indicated ascending aortic dissection with the involvement of the sinus of Valsalva (the sinus of Valsalva aneurysm) and cardiac tamponade. The patient was hemodynamically unstable and referred for urgent surgery.

**Figure 2:**
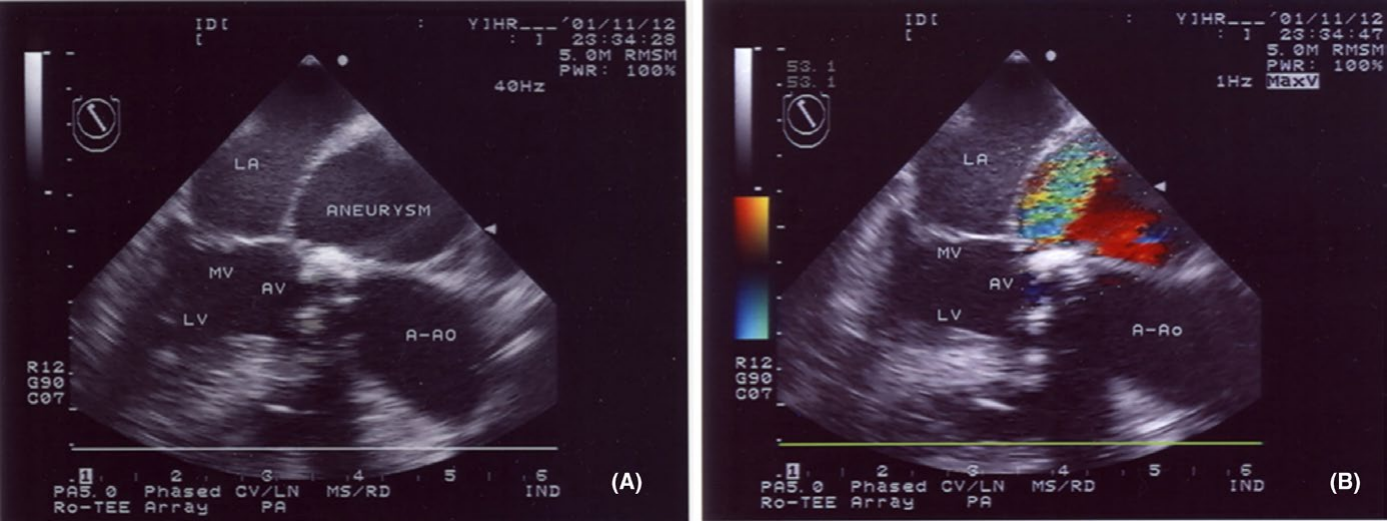
A, Transesophageal echocardiographic image (long‐axis view) of the left ventricular outflow tract showing a left ventricular aneurysm adjacent to the left atrium. B, Transesophageal echocardiographic image, same view as in image A, but with color‐flow Doppler added. Flow is clearly seen from the left ventricle to the aneurysm. A‐Ao, ascending aorta; AV, aortic valve; LA, left atrium; LV, left ventricle; MV, mitral valve

**Figure 3:**
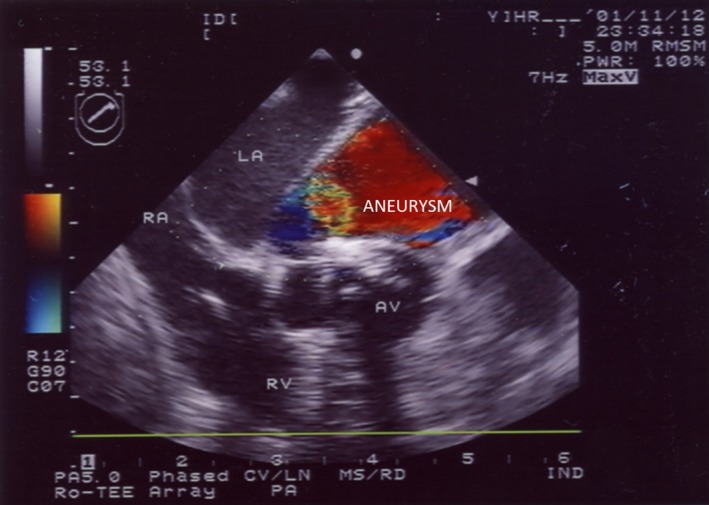
Transesophageal echocardiographic image (short‐axis view) of the aortic valve showing a left ventricular aneurysm lateral to the left atrium and calcified bicuspid aortic valve. AV, aortic valve; LA, left atrium; RA, right atrium; RV, right ventricle

Intraoperatively, TEE revealed a large aneurysm adjacent to the left atrium (LA; Figure 2, Movie S1). The aneurysm arose between the mitral and aortic valve annuli and extended behind the ascending aorta. Color Doppler flow imaging demonstrated systolic flow into the aneurysm with the evidence of continuity with the LV (Figure 2, Movie S1). The aneurysm laterally extended to the LA. There was also a calcified bicuspid aortic valve (Figure 3, Movie S2). There was a clear aneurysmal sac, the wall of which was continuous with the surrounding myocardium and had a regular border with no projection into the aneurysmal cavity. Therefore, the patient was diagnosed as having a bicuspid aortic valve and a ruptured LV aneurysm with cardiac tamponade. These data were relayed to the surgical team. During surgery, the cardiac tamponade was relieved, the aneurysmal orifice was closed with a patch (via the annulus of the aortic valve), and the aortic valve was replaced with a mechanical valve. No dissection of the ascending aorta was identified. Postoperatively, TEE confirmed that there was no connection between the LV and the aneurysm (Movies S3 and S4), and the patient made an uneventful recovery. He was later discharged in a satisfactory condition.

## 3 DISCUSSION

Several mechanical complications are associated with acute myocardial infarction, including ventricular free wall rupture with or without ventricular septal rupture, papillary muscle rupture leading to acute severe mitral regurgitation, cardiogenic shock from extensive left or right ventricular infarction, and ventricular aneurysms. Among these, LV aneurysm is the most common mechanical sequela of acute myocardial infarction, occurring in approximately 15% of such cases (range, 3%‐38%).^1^, ^2^ Most cases of cardiac rupture after an acute myocardial infarction involve the free ventricular wall, and they are typically associated with sudden death due to the resulting hemopericardium and cardiac tamponade.^3^

In our case, we diagnosed the patient as having a true LV aneurysm rather than a pseudoaneurysm on the basis that it clearly communicated with the LV and was continuous with the myocardial wall throughout. The most likely cause of LV aneurysm in this case was probably a previous asymptomatic myocardial infarction, which could be explained by the patient's history of diabetes mellitus.

The posterolateral site of the aneurysm is also uncommon. Although TTE plays a useful role in the diagnosis of LV aneurysms,^4^, ^5^, ^6^ it can fail to detect those located posteriorly because of limited access to the posterior ventricular wall. In contrast, the proximity of the esophagus to the posterior ventricular wall makes TEE ideal for detecting posterior aneurysm.^7^, ^8^, ^9^ TEE during the cardiac surgery can provide essential diagnostic information, as has been reported in 12.8%‐38.6% of previous cases, with it even changing management in 4.4%‐14.6% of cases.^10^

In summary, this case report showed that intraoperative TEE is useful for obtaining a correct diagnosis of ruptured LV aneurysm that is missed by preoperative TTE. By providing more accurate information when assessing posterior ventricular aneurysms, TEE is invaluable when intraoperatively developing surgical plans. This may particularly be true for cases in which there has been an insufficient preoperative assessment in urgent settings.

## CONFLICT OF INTEREST

None declared.

## AUTHOR CONTRIBUTION

TT: drafted the article and involved in approval of article. SS: involved in critical revision of article, approval of article, and data collection.
